# The Effect of Different Amounts of Cinnamon Consumption on Blood Glucose in Healthy Adult Individuals

**DOI:** 10.1155/2019/4138534

**Published:** 2019-03-04

**Authors:** Nildem Kizilaslan, Nihal Zekiye Erdem

**Affiliations:** ^1^Istanbul Medipol University Institute of Health Sciences, Department of Nutrition and Dietetics, Turkey; ^2^Istanbul Medipol University School of Health Sciences, Department of Nutrition and Dietetics, Turkey

## Abstract

**Background:**

This study was aimed at investigating the effect of consumption of different amounts of cinnamon on preprandial blood glucose (PrBG), postprandial blood glucose (PoBG), glycosylated hemoglobin (HbA1c), and body mass index (BMI).

**Methods:**

This study was carried out on 41 healthy adult individuals. The individuals were divided into 3 groups and monitored for 40 days. The first, second, and third groups were given 1 g/day, 3 g/day, and 6 g/day cinnamon, respectively. Before the beginning of the consumption of cinnamon, HbA1c and PrBG blood tests of the individuals were examined on an empty stomach at family practice centers. Two hours after these tests were carried out and breakfast, PoBG tests were performed.

**Results:**

According to the findings of the study, the differences between the average weight measurements, BMI values, and HbA1c values before consumption on days 20 and 40 were not statistically significant in the individuals consuming 1 g, 3 g, and 6 g of cinnamon a day. The difference between the average PrBG measurements was found to be significant in the individuals consuming 6 g of cinnamon per day. The difference between the average PoBG measurements before consumption on days 20 and 40 was significant in the individuals consuming 1 g, 3 g, and 6 g of cinnamon per day.

**Conclusions:**

In particular a 3–6 g of cinnamon consumption was found to affect certain blood parameters of individuals positively. Therefore, it is considered to be beneficial to raise awareness of individuals to be conscious to regularly consume cinnamon.

## 1. Introduction

The most common metabolic disease in the world is reported to be type 2 diabetes. It is estimated that this disease will rise to 366 million by 2030 [1]. The cause of type 2 diabetes is considered to be multifactorial. However, it is stated that nutrition has a significant role in the disease's turning into a chronic disease [2].

The chemical content of cinnamon species is seen to be different from each other. Accordingly, while the Chinese cinnamon has 85–90% cinnamaldehyde, this ratio is 65–70% in the Ceylon cinnamon. Moreover, this ratio varies according to the quality of the cinnamon. Studies have shown that ground cinnamon is more effective than its extract. It has been reported in clinical studies that Chinese cinnamon is more effective than Ceylon cinnamon [3].

Today, it is seen that research studies arguing that cinnamon can be used against diabetes, which has become an important health problem, have increased.

One of the most discussed effects of cinnamon has been its effect of regulating individuals' insulin resistance and preprandial blood glucose [4–6]. Cinnamon is also claimed to be a natural insulin stimulant [7, 8]. The natural agents found in cinnamon serve as insulin to keep the blood glucose level stable [8].

Cinnamon exhibits characteristics that mimic insulin, such as the activity of biologically active substances to activate insulin receptor kinase, increasing glucose uptake, autophosphorylation of the insulin receptor, and glycogen synthase activity [9]. It has been stated that cinnamon increases glycogen storage by affecting the glycogen synthesis activity [7]. In a study, it was found that the cinnamon peel extract would increase insulin sensitivity and raise glucose intake [10]. Water-soluble components of cinnamon have been found to enhance the effectiveness of the insulin signaling pathway [11].

There is evidence that cinnamon provides glucose regulation. It is not known for sure whether it controls the type 2 diabetes mellitus [12]. Procyanidin type-A polymers found in cinnamon are stated to improve insulin receptor autophosphorylation and, thus, show their effect by increasing the sensitivity to insulin [13].

It has been shown that cinnamon, which is rich in polyphenolic components, reduces oxidative stress and corrects impaired preprandial glucose if consumed 500 mg/dl a day for 12 weeks [14].

Studies that examine the effects of cinnamon on individuals are mostly focused on individuals who are not healthy. However, literature reviews show that studies on the effects of cinnamon consumption on healthy individuals are quite insufficient. This study was aimed at investigating the effect of consumption of different amounts of cinnamon on PrBG, PoBG, HbA1c, and BMI in healthy individuals.

## 2. Material and Method

### 2.1. Research Agenda and Sample

The study was conducted as a randomized-controlled clinical trial at the family practice centers, Tokat/Turkey. This study was carried out between March 11 and April 25, 2016, on healthy and voluntary adult individuals. Healthy and voluntary 41 adult people participated in the study. The participants were divided into 3 groups according to the amounts of their daily cinnamon consumption. The research was completed with 14 people in group 1, 14 people in group 2, and 13 people in group 3. Individuals without chronic disease and not using drugs were included in the study. Individuals with an allergy to cinnamon, patients with symptoms suggestive of peptic ulcer disease and those with a past history of peptic ulceration, patients with chronic illness, and individuals using medical drugs were not included in the study. This study was conducted according to the guidelines laid down in the Declaration of Helsinki and all procedures involving human subjects/patients were approved by the Istanbul Medipol University Non-Interventional Clinical Researches Ethical Council. All persons gave their informed consent prior to their inclusion in the study. This research did not receive any specific grant from funding agencies in the public, commercial, or not-for-profit sectors.

### 2.2. Data Collection

The individuals in the first, second, and third groups who participated in the study were given 1 g/day, 3 g/day, and 6 g/day cinnamon, respectively. Cinnamon was prepared completely by the researcher. When cinnamon was prepared by the researcher, its freshness, shelf life, quality, lack of additives, storage conditions, and shape were taken into consideration.* Cinnamomum cassia* type cinnamon peel was brought to an herbalist to specially have it ground. By considering the amounts of consumption, 1 g, 3 g, and 6 g ground cinnamon bags were prepared for the individuals in each group. Each person was given 40 bags of ground cinnamon depending on the amount he or she used. The participants were recommended to take the cinnamon by mixing it with some apple and milk. It was explained to the individuals what would be done after the 40th day of the 21–40 days of cinnamon consumption. Body weights (kg) and heights (cm) of the individuals were measured by the researcher before they begun to consume cinnamon. A portable stadiometer device was used to measure the heights. Body weight measurements were carried out by using a +/- 100 g precision digital scale. The body mass indices (BMI) of the individuals were calculated with the following formula.

(1) $$\begin{array}{c} BMI\;\left(\;kg/m^{2}\;\right)=\frac{\text{Body Weight}\;\left(kg\right)}{{\left(Height\;\left(m\right)\right)}^{2}} \end{array}$$

Messages were sent to each individual personally every day in order to inform and remind them, and a mailing group was created. Through this formation, it was ensured that they consumed cinnamon every day. Before the beginning of the consumption of cinnamon, after fasting for at least 10 hours, preprandial blood glucose (PrBG) was obtained from the subjects in the morning. Two hours after these tests were carried out and breakfast, postprandial blood glucose (PoBG) tests were performed. Blood samples concentration in the serum was determined by the hexokinase method. HbA1c was measured by high-performance liquid chromatography. The serum samples were centrifuged for 10 minutes at a speed of 3000 rpm before the analyses. The same procedures for preprandial blood glucose (PrBG) and postprandial blood glucose were repeated on days 20 and 40 after the start of consumption. At the same time, the same procedure for HbA1c was repeated on day 40 after the start of consumption. The study was registered in family practice centers.

### 2.3. Statistical Methods Performed for Data Analysis

The SPSS 20.0 package program was used for the statistical analyses of the study. In statistical analyses, the data were expressed through descriptive values, arithmetic means ± standard deviations, minimum and maximum values, frequencies, and percentages. The chi-square test was used when comparing two or more independent groups in categorical variables, and the normality of the numerical variables was tested using the Shapiro-Wilk test [15, 16]. Parametric paired (dependent) samples t-tests were carried out for the data with normal distribution in two dependent groups, and parametric one-way analyses of variance for repeated measures were used for the data with normal distribution in more than two groups. The analysis results were interpreted by evaluating at 95% confidence interval and p<.05 significance level.

## 3. Results

### 3.1. Certain Demographic Characteristics of Individuals

The distribution according to gender and age groups of the individuals consuming cinnamon is given in Table 1. Of the individuals voluntarily participating in the study, 46.34% were female and 53.66% were male. The average age of the individuals was 37.95±8.40 years. Of the individuals, 34.14% were in the age group of 24–35, 39.02% were in the age group of 35–45, and 26.84% were in the age group of 46 and above. There was no statistically significant difference between the levels of cinnamon consumption according to gender and age groups of the individuals (p>.05).

### 3.2. Height, Weight, and Anthropometric Measurements of the Cinnamon-Consuming Individuals by Gender

The distribution of the cinnamon-consuming individuals according to their anthropometric measurements is given in Table 2. Proportionally, there was no significant change between the preconsumption and postconsumption average body weights of the individuals consuming 1 g, 3 g, and 6 g of cinnamon. Based on the analysis, there was no statistically significant difference between the average body weight measurements on days 20 and 40 before the start of the consumption of cinnamon (p>.05). There were no proportional differences between the BMI averages before and after consuming cinnamon. There was no statistically significant difference between the average BMI measurements on days 20 and 40 before the start of the consumption of cinnamon (p>.05).

### 3.3. Analysis of the Effect of Cinnamon on Blood Glucose

#### 3.3.1. Preprandial Blood Glucose (PrBG) Level

Table 3 shows the effect of cinnamon on preprandial blood glucose levels of the individuals.

Compared to the initial measurements of average preprandial blood glucose levels of the individuals consuming cinnamon, there were decreases in averages on days 20 and 40, specifically, 4.17% and 4.90% in those consuming 1 g of cinnamon per day, 1.84% and 2.75% in those consuming 3 g of cinnamon per day, and 4.55% and 5.92% in those consuming 6 g of cinnamon per day, respectively. There was no statistically significant difference between the average preprandial blood glucose measurements before the consumption of cinnamon on days 20 and 40 in the individuals consuming 1 g and 3 g of cinnamon per day (p>.05). However, there was a statistically significant difference between the average preprandial blood glucose measurements before the consumption of cinnamon on days 20 and 40 in the individuals consuming 6 g of cinnamon per day (p<.05). The significant difference was found to be between the average preprandial blood glucose level before consumption (1) and the average preprandial blood glucose level on day 40 (3). Accordingly, the average preprandial blood glucose level measured after day 40 showed a significant decline compared to the average preprandial blood glucose level before consumption.

#### 3.3.2. Postprandial Blood Glucose (PoBG) Level

Table 4 shows the effect of cinnamon on postprandial blood glucose levels of the individuals.

Compared to the initial measurements, an average of 1.51% of decline was observed on day 20 of the cinnamon consumption in the postprandial blood sugar levels of the individuals consuming 1 g of cinnamon per day, and an average of 8.48% decline was observed on day 40. Statistically significant differences were found between the average postprandial blood glucose levels of the individuals on the initial day, on day 20 and day 40 (p<.05). The significant difference was found to be between the average postprandial blood glucose level on day 20 (2) and that on day 40 (3). Accordingly, the average postprandial blood glucose level measured after day 40 showed a significant decline compared to the average postprandial blood glucose level on day 20.

Compared to the initial measurements, an average of 6.47% of decline was observed on day 20 of the cinnamon consumption in the postprandial blood sugar levels of the individuals consuming 3 g of cinnamon per day, and an average of 10.7% decline was observed on day 40. The postprandial blood glucose of the individuals consuming 6 g of cinnamon per day decreased similarly by 3.55% and 12.71%, respectively. There was a statistically significant difference between the average postprandial blood glucose measurements before the consumption of cinnamon on days 20 and 40 in the individuals consuming 3 g and 6 g of cinnamon per day (p<.05). The significant difference was found to be between the average postprandial blood glucose level before the beginning of cinnamon consumption (1) and that on day 40 (3). Accordingly, the average postprandial blood glucose level measured after day 40 showed a significant decline compared to the average postprandial blood glucose level before the consumption of cinnamon.

#### 3.3.3. HbA1c (Glycosylated Hemoglobin) Level

Table 5 shows the effect of cinnamon on the HbA1c levels of the individuals.

Compared to the initial measurements, 0.20% decline was observed on day 40 of the cinnamon consumption in the average HbA1c levels of the individuals consuming 1 g of cinnamon per day, 0.19% decline in those consuming 3 g per day, and 1.90% decline in those consuming 6 g per day. There was no statistically significant difference between the average HbA1c level measurements before consumption and on day 40 in the individuals consuming 1 g, 3 g, and 6 g of cinnamon per day (p>.05).

## 4. Discussion

Considering the studies on individuals, it is seen that there are positive effects of cinnamon consumption in healthy individuals, although they have differences. Predominantly the studies on individuals who are not healthy attract attention in both the national and the international literature. Studies on healthy individuals were observed to be very limited. In this regard, studies on the effects of cinnamon consumption of healthy individuals on blood glucose, as well as studies on nonhealthy individuals, were covered. The main aim of this approach was to observe the effects of cinnamon consumption not only on individuals who are healthy but also on individuals who are not healthy, in terms of the blood parameters that were addressed.

In a study on healthy individuals, 3 different oral glucose tests were administered to 7 healthy individuals. Accordingly, the individuals have consumed 5 g of placebo, 5 g of cinnamon, and 5 g of cinnamon 12 hours after the oral glucose test. In the group that consumed cinnamon, there was a significant decline in the total plasma glucose response, and insulin sensitivity developed [17]. In another study of the same researchers on healthy individuals, they have found that cinnamon has made improvements in glucose and insulin sensitivity during 14-day periods [18].

In the study of Tang et al., it was found that there was no change in preprandial blood glucose and blood lipids at the end of 4 weeks in healthy individuals who were given cinnamon [19].

It was found also in the present study that different levels of cinnamon consumption caused a decrease in preprandial blood glucose levels, although small in magnitude. It was seen that there was also a statistically significant difference between the average preprandial blood glucose measurements before beginning the consumption of cinnamon and on day 40 in the individuals consuming 6 g of cinnamon per day.

In the study of Kim et al., hydroxycinnamic acid was obtained by refining from cinnamon. They investigated this acid as an antidiabetic derivative. They found that it had the highest glucose transport activity. They determined that it reduced the plasma glucose by improving glucose transport [20]. In a study of obese and normal weight individuals, in the measurements made 120 minutes after cinnamon consumption, cinnamon was found to reduce the postprandial blood glucose in both groups [21].

It was seen also in the present study that there were proportional declines in the postprandial blood glucose levels of the individuals consuming 1 g, 3 g, and 6 g of cinnamon compared to the initial measurements and that there were statistically significant differences, as well.

In a placebo-controlled study on 60 volunteer patients over the age of 40, the volunteers were given 1 g, 3 g, and 6 g of ground cinnamon after meals for the first 40 days, and a placebo treatment was administered for the next 20 days. Serum glucose levels dropped by 18–29%. The supplementation of 1 g of ground cinnamon per day was found to improve preprandial blood glucose and blood lipid profile. There was no significant change in the amount of ground cinnamon supplementation in the placebo group [7]. Because the target group was composed of diseased individuals, the study has shown that cinnamon causes significant positive proportional changes in the blood glucose profile based on the implementation. This may be attributed to the severity of the diseased patients' impaired preprandial blood glucose levels.

However, as in the present study, the decline in the healthy individuals remained proportional at lower levels. This can be explained by the fact that preprandial blood glucose levels are within the normal range in healthy individuals. Therefore, it is possible to say that cinnamon consumed at certain amounts contributes highly positively to the impaired preprandial blood glucose levels of diseased individuals whereas it mainly plays a regulating role in the preprandial blood glucose levels in healthy individuals. As a matter of fact, there was no significant change in preprandial blood glucose and lipids in the placebo group in the present study that was carried out, which confirms this thought. Therefore, in studies conducted on individuals who are not healthy, when there are significant declines, the effect of drugs should not be overlooked, considering that patients use drugs with effects that lower their blood glucose levels.

It has been reported in a study that 500 mg of cinnamon capsule per day provides positive improvement in the preprandial plasma glucose level of individuals diagnosed with metabolic syndrome [22].

In another study carried out to investigate the effect of consuming cinnamon at different levels on blood glucose, 30 patients with type 2 diabetes were divided into three groups of 10 people. The patients in each group were given 1 g, 3 g, and 6 g cinnamon capsules per day for 40 days. Blood glucose levels of the patients included in the study were measured at the beginning, on the 20th day, and on the 40th day, after consumption. According to the results of the study, it was determined that cinnamon lowered the blood sugar of patients distinctly and significantly [6].

In another study, 146 studies (clinical, in vivo, and in vitro) conducted until 2010 were identified and examined by selecting 30 clinical trials. From among these clinical trials that were examined, 8 studies with the desired characteristics were evaluated. Ultimately, it was seen that, in all studies demonstrating positive results, “Chinese cinnamon” was used. According to the results of that study, it has been emphasized that the amount and duration of use is important in order to achieve the effect. Accordingly, it has been suggested that at least 1–2 grams of ground or extract Chinese cinnamon should be used 1–2 months in order to see a minimal impact. Again, based on research results, it has been shown that although it causes no effect on blood glucose of people with normal blood glucose levels, it is effective on blood glucose in people with type 2 diabetes and prediabetes [23].

In the study of Stoecker et al., 137 type 2 diabetes mellitus patients were evaluated for 2 months. The use of 500 mg cinnamon capsules was found to cause a decrease in preprandial and postprandial blood glucose levels [24].

Compared to the initial measurements of average postprandial blood glucose levels of the individuals consuming cinnamon, there were declines also in the present study in averages on days 20 and 40—specifically, 1.51% and 8.48% in those consuming 1 g of cinnamon per day, 6.47% and 10.7% in those consuming 3 g of cinnamon per day, and 3.55% and 12.71% in those consuming 6 g of cinnamon per day, respectively. The decrease in the postprandial blood glucose of the individuals who consumed 6 g was more than those of the other groups.

In the study of Crawford et al., 109 type 2 diabetes mellitus (HbA1c>7) patients were evaluated for 90 days. It was found that the daily consumption of 1 g of cinnamon capsules significantly reduced the HbA1c level. In the group that used cinnamon, a 0.83% decrease was observed in the HbA1c value. The HbA1c value in the control group decreased by 0.37% at the end of 3 months [25].

In the study of Akilen et al., 2 g/day* Cinnamomum cassia* type cinnamon consumption for 12 weeks was observed to cause a significant decline in HbA1c level [26].

In a study of 5 prospective-controlled trials by Baker et al., cinnamon consumption was found to not alter preprandial blood glucose, HbA1c, and lipid parameters in patients with type 1 and type 2 diabetes [9].

A research study in Tabriz, Iran, was carried out on 60 patients with type 2 diabetes. Of these 60 patients, 30 people were in the experimental group, and 30 people were in the control group. In the study, the experimental group was given 1.5 grams of cinnamon a day, while the control group was given a capsule as a placebo with no effect on diabetes. According to the results of the study, the preprandial blood glucose and HbA1c did not have a significant difference in the control group. This difference was found to be significant in the experimental group (p<.05). It was found also in this study that cinnamon caused positive effects on preprandial blood glucose levels as well as HbA1c levels in patients with type 2 diabetes [27].

Another research study in Yazd, Iran, was carried out on 61 patients with type 2 diabetes. Of these 61 patients, 31 people were in the experimental group, and 30 people were in the control group. The study continued for 6 weeks. The experimental group was given 2 g of cinnamon per day (2 500 mg capsules every 12 hours). The control group was given capsules as a placebo that had no effect on diabetes. At the end of the study, there was no significant difference between the blood glucose and HbA1c values of the experimental and control groups [28].

In the study of Lu et al., a group that consumed ground cinnamon was compared with a placebo group. The study included 66 Chinese people with type 2 diabetes mellitus. At the end of 90 days, a significant decline was observed in HbA1c. No significant decline was observed in the placebo group. Preprandial blood glucose was found to decline significantly in both groups [29].

A meta-analysis of 6 clinical trials involving cinnamon has included 435 people. It was found that cinnamon reduced preprandial blood glucose, and HbA1c decreased in short-term studies [30]. In another study, 43 diabetes patients with an average HbA1c level of 7.1% were given 1 g of cinnamon per day for 3 months. It was reported that there was no change in preprandial blood glucose and HbA1c levels [31].

In the present study, although there were proportionally small changes in HbA1c levels at different levels of consumption, they were not significant. In all three groups, there was no statistically significant difference between the average HbA1c level measurements before consuming cinnamon and on day 40.

Research studies examining the effects of cinnamon on blood sugar of animals were found during the literature review. In a study, in which the effect of the cinnamon on insulin resistance and body composition was examined, 22 male Wistar mice were fed with a high-fat and high-fructose diet. A total of 20 g of cinnamon per kilogram was given with a high-fat and high-fructose diet. It was found that insulin sensitivity decreased, and body composition changed in the mice that were fed [32].

Kannappan et al. carried out a study on male Albino mice, dividing them into two groups: a control group and a group consuming cinnamon along with a high-fructose diet. A glucose tolerance test was administered. In mice with a high-fructose diet, glucose tolerance was improved. No significant changes were found in low doses [33].

In a study carried out by Qin et al, cinnamon components were added to the control group's drinking water to observe whether the cinnamon components increased the glucose use of male Wistar mice. A high-fructose diet was administered to the control and experimental groups for 3 weeks. The consumption of cinnamon components was found to prevent the development of insulin resistance in mice with high-fructose diet in the control group [34].

In their study, Taher et al. found that water-soluble cinnamon polyphenols developed adipogenesis [35]. In another study, cinnamon was found to activate insulin-induced glucose use in the epididymal adipose tissue in mice. Thus, they have found that it improves the glucose and insulin metabolism [5].

In in vivo studies, plasma glucose and insulin concentrations of mice were examined.* Cinnamomum cassia* was found to be more effective than* Cinnamomum zeylanicum* and reduced glucose levels in the blood glucose tolerance test [36].

In a study investigating the antidiabetic effect of* Cinnamomum cassia*, cinnamon was given to animals with type 2 diabetes mellitus for 6 weeks. Glucose intensity in the blood was found to be reduced significantly in this period [37].

Streptozotocin was given to diabetic Wistar mice for 45 days to examine the components of* Cinnamomum zeylanicum* exhibiting an antidiabetic effect, and cinnamaldehyde was administered. Plasma glucose concentration has decreased significantly compared to the control group. Moreover, the administration of cinnamaldehyde has lowered the HbA1c level [38].

In another study, mice were given cinnamon oil (25.5 mg/kg and 100 mg/kg) for 35 days. In the group receiving 100 mg/kg cinnamon oil, the preprandial plasma glucose level was found to be significantly reduced compared to that in the control group. Additionally, healing was observed in pancreatic *β* cell islets [39].

A study was carried out in Jordan on 75 patients with type 2 diabetes for 4 weeks; the patients were asked to take 2 g of ground cinnamon (two 500 milligrams of cinnamon capsules) immediately after breakfast, lunch, and dinner; and, as a result, a daily dose of 6 g of cinnamon was found to be effective in reducing blood glucose in a short time [40].

In a parallel study on patients with type 2 diabetes after menopause, the patients were given cinnamon (*Cinnamomum cassia*, 1.5 g/day) and placebo supplements for 6 weeks. Based on the study, no change was detected in preprandial blood glucose, preprandial insulin and HbA1c levels, blood lipids, and whole-body insulin resistance/sensitivity [41]. In another study, 25 postmenopausal women were examined. The consumption of 1500 mg of cinnamon per day has been observed not to reduce blood sugar compared to placebo [42]. In another study, 58 postmenopausal women with type 2 diabetes mellitus were evaluated. It was observed that consuming capsules containing 500 mg of cinnamon 2 times a day for 3 months had no significant effect [43].

## 5. Conclusion

It has taken place in both national and international literature that cinnamon reduces blood glucose in nonhealthy individuals, and many studies have been carried out on this subject. However, studies demonstrating the effect of cinnamon on the blood glucose of healthy individuals are little if any. In this study, it has been proven that cinnamon causes positive changes in the blood glucose levels of healthy individuals. In healthy individuals, the effects of cinnamon on blood glucose are positive but are in a way that is regulatory and to keep the blood glucose within the normal values/limits. Cinnamon led to significant changes in certain blood parameters examined at different consumption levels in both proportional and statistical terms. However, it can be said that this change differed depending on the daily consumed amount and that the differentiation increased when the consumed amount was 3–6 g. For this reason, more detailed and long-term studies are needed for the use of cinnamon in healthy individuals. More beneficial results can thus be achieved by enriching the data on the effects of cinnamon on healthy individuals.

## Figures and Tables

**Table 1:** Distribution of the cinnamon-consuming individuals by gender and age groups.

cinnamon consumption levels of individuals (g/day)									
	1g		3g		6g		Total		P^*∗*^

Gender	Number	%	Number	%	Number	%	Number	%	0.80

Women	6	42.86	6	42.86	7	53.85	19	46.34	

Men	8	57.14	8	57.14	6	46.15	22	53.66	

Total	14	100.00	14	100.00	13	100.00	41	100.00	

Age groups	36.21±8.40		37.50±6.54		40.31±10.14		37.95±8.40		0.09

24-35	6	42.86	4	28.57	4	30.77	14	34.14	

36-45	4	28.57	9	64.28	3	23.08	16	39.02	

46+	4	28.57	1	7.15	6	46.15	11	26.84	

Total	14	100.00	14	100.00	13	100.00	41	100.00	

^*∗*^The chi-square test was used when calculating the p values.

**Table 2:** Distribution of the cinnamon-consuming individuals according to their anthropometric measurements.

cinnamon consumption levels of individuals (g/day)						
	1g		3g		6g	

	Women	Men	Women	Men	Women	Men

Height (cm)	165.16 ±7.54	177.00±8.07	162.16±6.17	176.75±5.17	162.57 ±5.15	173.66±6.34
Mean±SD	171.92 ±9.69		170.50±9.22		167.69 ±7.95	

Weight (kg)						

Body Weight1^*∗*^	62.63±11.92	83.25±7.78	61.71±5.80	80.47±7.40	64.61±10.49	81.50±10.30
Mean±SD	74.41±14.12		72.43±11.63		72.40±13.26	

Body Weight2^*∗*^	62.75±11.90	82.26±7.78	62.11±5.81	80.11±7.45	64.57±10.23	81.21±9.73
Mean±SD	73.90±14.05		72.40±11.32		72.25±12.90	

Body Weight3^*∗*^	62.08±11.98	82.90±8.58	62.45±6.17	80.12±7.62	64.08±9.93	81.11±9.63
Mean±SD	73.97±14.46		72.55±11.32		71.94±12.88	

P^*∗∗∗*^	0.383		0.881		0.371	

BMI (kg/m^2^)						

BMI 1^*∗∗*^	22.96±4.38	26.56±1.89	23.28±1.94	25.78±2.36	24.58±4.55	26.90±1.86
Mean±SD	25.02±3.56		24.71±2.47		25.65±3.63	

BMI2^*∗∗*^	23.00±4.17	26.25±2.06	23.63±1.91	25.70±2.40	24.55±4.40	26.80±1.82
Mean±SD	24.85±3.43		24.81±2.37		25.59±3.52	

BMI 3^*∗∗*^	22.76±4.35	26.48±2.06	23.46±1.96	25.61±2.50	24.32±4.24	26.73±1.81
Mean±SD	24.89±3.63		24.69±2.91		25.43±3.45	

P^*∗∗∗*^	0.477		0.780		0.134	

^*∗*^Body Weight 1: Before Consumption; Body Weight 2: Day 20; Body Weight 3: Day 40

^*∗∗*^BMI1: Before Consumption; BMI2: Day 20; BMI3 Day 40

^*∗∗∗*^One-way ANOVA for Repeated Measures was used when calculating the p values.

**Table 3:** Effect of cinnamon on preprandial blood glucose level.

preprandial blood clucose (mg/dl)					
Consumption Levels (g/day)	before consumption (1)	on day 20 (2)	on day 40 (3)	P^*∗*^	Result

1g	92.37±4.44 (86.00-101.50)	88.52± 7.21 (78.10-101.00)	87.84±10.88 (65.00-106.00)	0.128	not significant

3g	85.92±12.78 (73.00-122.00)	87.50± 9.77 (70.00-101.00)	83.56± 6.01 (74.00-94.00)	0.438	not significant

6g	94.66±6.37 (87.00-108.00)	90.35± 10.97 (73.00-107.00)	89.06± 9.24 (74.90-102.00)	*0.035*	* significant(1-3)*

^*∗*^One-way ANOVA for Repeated Measures was used when calculating the p values.

**Table 4:** Effect of cinnamon on postprandial blood glucose level.

postprandial blood glucose (mg/dl)					
Consumption Levels (g/day)	before consumption (1)	on day 20 (2)	on day 40 (3)	P^*∗*^	Result

1g	93.94±19.12 (62.40-126.00)	92.52± 11.77 (74.90-123.00)	85.97±11.12 (63.60-108.00)	*0.028*	*significant(2-3)*

3g	96.97±23.86 (58.00-151.00)	90.69± 17.61 (61.00-119.00)	86.52± 13.84 (63.60-108.00)	*0.018*	*significant(1-3)*

6g	105.67±22.46 (76.00-146.00)	101.92± 23.83 (77.00-162.00)	92.23± 14.24 (78.00-128.00)	*0.017*	*significant(1-3)*

^*∗*^One-way ANOVA for Repeated Measures was used when calculating the p values.

**Table 5:** The effect of cinnamon on the HbA1c level.

HbA1c level (%)				
Consumption Levels (g/day)	before consumption (1)	on day 40(3)	P^*∗*^	Result

1g	4.98 ±0.49 (4.20-5.60)	4.97± 0.36 (4.20-5.60)	0.948	not significant

3g	5.08 ±0.32 (4.41-5.70)	5.07± 0.26 (4.40-5.40)	0.909	not significant

6g	5.26 ±0.38 (4.70-6.10)	5.16± 0.36 (4.70-5.70)	0.542	not significant

^*∗*^Paired samples t-tests were used when calculating the p values.

## Data Availability

No data were used to support this study.
